# On the Cow-Pox

**Published:** 1799-03

**Authors:** John Sims


*The ſignature of the following communication, on this ſubject, renders any introductory obſervations unneceſſary.*

### *To the Editors of the Medical and Phyſical Journal.*

THE following extract of a letter from an intelligent gentleman, not of the profeſſion, appearing to me to deſerve the attention of medical practitioners, and particularly of thoſe who ſeem inclined to recommend the general inoculation of the cow-pox, as a preſervative from the ſmall-pox, I communicate it, with the hope that it will be inſerted in the firſt number of the Medical aud Phyſical Journal.

" There is a gentleman of eminence in the law, now living in Briſtol, who has had the cow-pox twice, and being afterwards inoculated for the ſmall-pox, had it in ſo great abundance that his life for ſome time was deſpaired of: he deſcribes the cow-pox as the moſt loathſome of diſeaſes, and adds that his right arm was in a ſtate of eruption both the firſt and ſecond time from one extremity to the other; the pain was exceſſive and his fingers ſo ſtiff he could ſcarcely move them. At a time when parents are beginning to talk ſeriouſly of inoculating their children with this loathſome and uſeleſs diſeaſe, the above above ſtatement may not be unacceptable. The gentleman alluded to was the ſon of a farmer who kept ſeventy cows, of which he, being then a lad, milked eighteen himſelf; they were all of them infected with this diſorder at one time; he caught it, and ſuch was the abhorence it created in the family, that they made no uſe of the milk as long as it laſted. He never heard, nor does he believe, that this complaint in cows originates, as ſuppoſed by Dr. Jenner, from any communication with that acrid humour, called *the greaſe in horſes.*"

What this gentleman remarks of the loathſomeneſs of the diſeaſe, although a circumſtance entirely overlooked in Dr. Jenner's account, appears to be in itſelf a formidable objection to its introduction, even ſhould it be found to anſwer the purpoſe for which it has been recommended. But, if in one caſe, and that where the patient has been twice ſo ſeverely afflicted with it, it has already been found to be ineffectual in preſerving from the infection of the ſmall-pox, it will ſurely make us heſitate in recommending the introduction of a hitherto nearly unknown diſeaſe. At firſt ſight, indeed, it ſhould ſeem very improbable that a diſeaſe, which has not the power of preſerving the conſtitution from a ſecond attack, ſhould be capable of making ſuch a change in it, as to enable it to reſiſt the infection of a ſomewhat analogous, but far more violent diſeaſe; but the facts and experiments, brought forward by Dr. Jenner, ought certainly to call forth an inquiry, from ſuch medical practitioners as are ſo ſituated that they can have it in their power to make real obſervations on the diſeaſe, and its ſuppoſed preſervative powers; but I can by no means approve of making ſuch raſh experiments upon our fellow-creatures, as the inſertion by inoculation of a variety of acrid animal poiſons, the effects of which, upon the individual conſtitution, no man can, *a priori*, judge of, and who ſhall ſay that the individual only ſhall in all caſes be the ſufferer? If ſuch hazardous experiments be not diſcouraged, there is ſome reaſon to fear that to the opprobrium, the profeſſion already lies under, of not being able to cure many of the exiſting diſeaſes, will be added, that of having introduced new ones.

*New Bridge-ſtreet.* Feb. 13, 1799. *JOHN SIMS.*

---

*From the above general ſtatement, our readers will perceive that the great queſtion reſpecting the utility of the cow-pox is at iſſue before the public.*

*T. B.*

Remarks

